# Mesoscale Finite Element Modeling of Mortar under Sulfate Attack

**DOI:** 10.3390/ma15155452

**Published:** 2022-08-08

**Authors:** Zhongzheng Guan, Peng Wang, Yue Li, Yong Li, Bo Hu, Yichao Wang

**Affiliations:** 1 Key Laboratory of Roads and Railway Engineering Safety Control, Ministry of Education, Shijiazhuang Tiedao University, Shijiazhuang 050013, China; 2 School of Civil Engineering, Hebei University of Technology, Handan 056107, China; 3 Key Laboratory of Urban Security and Disaster Engineering of Ministry of Education, Beijing Key Laboratory of Earthquake Engineering and Structural Retrofifit, Beijing University of Technology, Beijing 100124, China

**Keywords:** mortar, sulfate attack, mesoscale FE model, ITZ, calibrated constitutive relations

## Abstract

In this paper, a 2D mesoscale finite element (FE) numerical model of mortar, considering the influence of the ITZ, was proposed to evaluate the corrosion of mortar in sodium sulfate. On the mesoscale, the corroded mortar was regarded as a three-phase composite material composed of sand, cement paste, and an interface transition zone (ITZ). Firstly, the volume fractions and mechanical parameters (elastic modulus, Poisson’s ratio, and strength) of the mesoscale phases were obtained. Then, the cement paste and the ITZ were combined to form an equivalent matrix by homogenization methods, and the calibrated constitutive relations of the equivalent matrix were established. Subsequently, a two-dimensional (2D) random circular aggregate (RCA) model and a 2D random polygonal aggregate (RPA) model of corroded mortar were established using the random aggregate model. The failure process of corroded mortar specimens under uniaxial compression was simulated by the mesoscale FE numerical model. Comparing the simulation results with the measured stress–strain curves of the uniaxial compression test, it was found that the simulation results of the 2D RP model were closer to the experimental results than those of the 2D RC model. Meanwhile, the numerical simulation results were in good agreement with the experimental results, and the error values of peak stress between the simulation results and the measured results were within 7%, which showed that the 2D mesoscale FE model could accurately predict the results of a uniaxial compression test of a mortar specimen under sulfate attack.

## 1. Introduction

Sulfate attack is an important factor affecting the main deterioration mechanisms of cement-based materials, which seriously influence the service performance and durability of concrete structures [1,2,3,4,5]. Many environments, such as seawater, brine, groundwater, decaying organic matter, and industrial effluent, contain a large amount of sulfate, which can significantly corrode concrete structures [6,7,8]. The study of the durability and mechanical properties of cement-based materials under external sulfate attack has become a major issue in structural engineering in recent decades [9,10,11,12,13]. As is well-known, the mortar scale is the midpoint between the cement scale and the concrete scale. Therefore, research into sulfate attack on the mortar level is of great significance.

In recent years, many scholars have conducted a great deal of research on the deterioration mechanisms of cement-based materials under external sulfate attack [14,15,16,17]. Sulfate ions enter into cement-based materials through microcracks and the micropore structure [10] and react with cement hydration products to produce ettringite (AFt) and other expansive products [16,17]. As the volume of expansive products is larger than the volume of the original components, the formation of expansive products generates expansion stress in the surrounding micropore structure [18]. The expansion stress leads to the generation and propagation of microcracks in the cement-based material when the expansion stress is greater than the tensile strength, which causes a change in the mechanical properties of the cement paste and ITZ in the mortar and leads to a change in the mechanical properties of the mortar [12,18,19]. Sulfate ions gradually enter the interior of the mortar from the outside in, and the deterioration in the mechanical properties of the mortar is a layer-by-layer process from the surface to the inside. Therefore, the sulfate ion erosion time and spatial distribution have an important impact on the mechanical properties of cement-based materials.

Cement-based materials are multi-scale and multi-phase composite materials. The changes in the material deformation, cracking, and mechanical properties on the macroscale are due to the changes in the material on the mesoscale. Many scholars have made remarkable contributions to our understanding of the mechanical properties of cement-based materials from the perspective of finite element simulations on the mesoscale. Considering the influence of pore structure and ITZ on concrete, Jin [20,21,22,23] used a mesoscale equivalent mechanical model to simulate the tensile and compressive behavior of concrete. Based on a random aggregate model, Leite [24], Wriggers [25], Stenfan [26], and Du [27] carried out a series of finite element numerical simulations to investigate the macroscopic mechanical properties of concrete, such as the uniaxial compression, uniaxial tensile, shear, and bending, and obtained superior calculation results. Qi [28] and Li [29] established 3D finite element models of concrete using reconstructed X-ray CT scanning images to achieve the numerical simulation of the compressive failure process. Li [30] numerically simulated and analyzed a uniaxial compression test of a cement paste specimen under sulfate attack using a 3D mesoscale finite element model created with the X-ray CT technique. However, there are few studies on the finite element numerical simulation of mortar specimens under sulfate attack.

The ITZ between the aggregates and the matrix has a significant influence on the macroproperties of the composites. The porosity and connectivity of the pore structures in the ITZ are much higher than those in the matrix [31]. The ITZ has the characteristics of a low strength, low elastic modulus, and high permeability [32,33]. The ITZ is the weakest link in all the phases of concrete materials. The macroproperties of concrete materials (such as the strength and elastic modulus) are largely related to the geometric and physical properties of the ITZ. Liang [34] considered the influence of the ITZ, divided concrete into four levels based on the results of the nanoindentation test, and calculated the elastic modulus of concrete using homogenization methods. Wu [35] studied the relationship between the ITZ and the damage evolution of concrete under the action of sodium sulfate erosion. Several researchers have conducted various microstructure assessments to evaluate the effective thickness of the ITZ, usually determining that the thickness of the ITZ is 15–50 μm [36]. Mondal [37,38] studied the elastic properties of the ITZ between fine aggregate and cement paste using nanoindentation technology. It was found that the measured elastic modulus of the indentation point in the ITZ gradually increased with the distance to the center of the fine aggregate, and its average value was 18 GPa, about 85% of the elastic modulus of the matrix. The results showed that the elastic modulus of the ITZ is significantly lower than that of cement paste. Therefore, the ITZ is a key factor for simulating the uniaxial compression process of a mortar specimen under sulfate attack.

**Figure 1:**
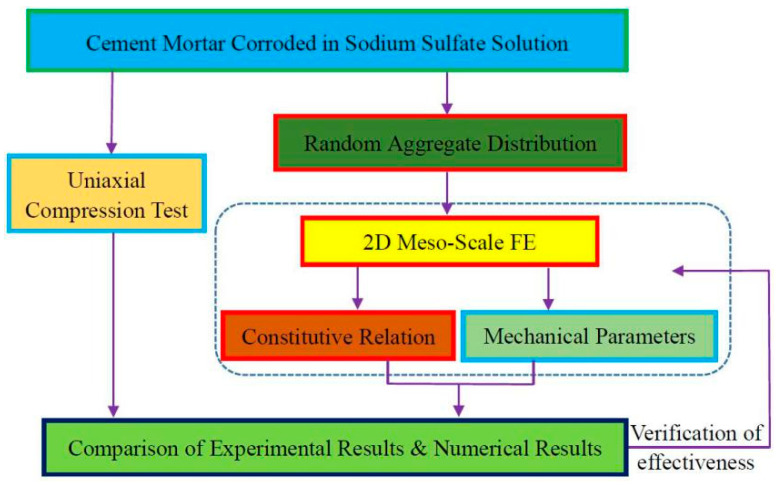
Roadmap of this paper.

In this paper, an experimental test and an FE numerical simulation were combined to investigate the uniaxial compressive failure process of mortar specimens corroded in sodium sulfate solution. Firstly, the mortar specimen was regarded as a three-phase composite of cement paste, sand, and ITZ on the mesoscale. Subsequently, a 2D mesoscale finite element (FE) model of corroded mortar was established using the random aggregate distribution model with calibrated constitutive relations and the mechanical parameters of the phases. Finally, the failure process of a corroded mortar specimen under uniaxial compression was simulated by the 2D mesoscale FE model. The simulation results were compared with the stress–strain curve measured by the uniaxial compression test to verify the correctness of the 2D mesoscale FE model. The roadmap of this paper is shown in Figure 1.

## 2. Materials and Test Methods

### 2.1. Materials and Samples

#### 2.1.1. Materials

In this study, P.I 52.5 Portland cement was selected for testing, and its mineral composition is shown in Table 1. The fine aggregate selected for testing was natural river sand with a fineness modulus of 2.45. The maximum particle size of fine aggregates was 4.75 mm, and the minimum particle size was 0.035 mm. The mud content (by mass) of fine aggregates was less than 1.5%. The cumulative percentage retained of fine aggregates is shown in Table 2.

Sodium sulfate (Na_2_SO_4_) was selected as the corrosion medium of the external sulfate solution with a sulfate ion mass concentration of 5% (molar concentration 352 mol/m^3^).

#### 2.1.2. Sample Preparation

In this study, two types of cement mortar sample, with water/cement ratios (w/c) of 0.53 (M1) and 0.35 (M2), were selected, and the mix proportions of the mortar samples are shown in Table 3. The size of the mortar samples was 20 mm × 20 mm × 40 mm. Mortar samples were kept under laboratory conditions (20 ± 5 °C) for 24 h before demolding, then cured for 28 d under standard curing conditions with a temperature of 20 °C and a relative humidity of 95%. Subsequently, mortar samples were soaked in a sealed container filled with Na_2_SO_4_ solution at 20 ± 5 °C. The Na_2_SO_4_ solution was replaced every 3 d to ensure a constant concentration of sulfate ions.

### 2.2. Test Methods

The uniaxial compression test of mortar samples was conducted using a microcomputer-controlled electro-hydraulic servo pressure testing machine (Zwick/Roell Z050, Ulm, Germany) with a maximum loading of 50 kN and a loading speed of 0.5 mm/min.

## 3. Random Placement and Generation of Fine Aggregates in Mortar

Fine aggregates play the role of a supporting skeleton in mortar and have a significant influence on improving its performance by reducing its shrinkage and cement consumption. The gradation, percentage, position, and geometry of fine aggregates have an important impact on the macro mechanical properties of mortar. Therefore, it was necessary to consider the generation and placement of fine aggregates with regard to gradation, distribution, and particle shape in order to establish a mesoscale model of mortar consistent with the actual situation. Firstly, the fine aggregate gradation was determined by the Fuller gradation curve, and then the geometric position of the fine aggregates in the mortar specimen was determined using the Monte Carlo method. Subsequently, according to the fine aggregate gradation and fine aggregate shape, the fine aggregate structure in the mortar specimen was simulated, and the random aggregate model of mortar was established.

### 3.1. Gradation Analysis of Fine Aggregates in Mortar

The gradation of fine aggregates in mortar mainly depends on two factors: the size of the aggregate particles and the quantity of the aggregates of each particle size. Aggregate gradation is very important to the mechanical properties of mortar. W.B. Fuller [39] proposed an ideal aggregate grading curve based on the maximum density theory. Through the reasonable arrangement of aggregate particle sizes, the optimum gradation with the highest compactness and the smallest surface area was obtained, which was widely recognized and applied. The Fuller grading curve [39] has a high degree of identity with the optimum grading obtained by the test, and its basic formula is:

(1) $$P(d)=100{\left(d/d_{\max}\right)}^{n}$$

where *P*(*d*) is the percentage of aggregate particle mass with a particle size less than *d* in the total aggregate mass; *d*_max_ is the maximum particle size of the aggregate; and *n* is the exponent of the equation, taken as 0.5.

According to the mix proportion of the mortar, Lu and Torquato’s model, and the thickness of the ITZ, the volume fractions of the fine aggregates in the mortar (M1 and M2) were 0.47 and 0.46, respectively [40]. As seen in Table 2, the volume proportions of fine aggregates with a particle size greater than 4.75 mm, less than 0.15 mm, or between 2.36 mm and 4.75 mm were very small. Considering the number and size of fine aggregates and the meshing of fine aggregates in finite element analysis, the fine aggregates with a particle size greater than 4.75 mm were rounded off, and the corresponding volume fraction was combined with that of the fine aggregates with a particle size of 4.75 mm–1.18 mm. Meanwhile, the fine aggregates with a particle size of less than 0.15 mm were also rounded off, and the corresponding volume fraction was combined with that of the fine aggregates with a particle size of 0.3 mm–0.15 mm. Subsequently, the mass ratio of the fine aggregates in each particle size range to the total aggregate was obtained using the Fuller formula. Then, the volume ratio of the fine aggregates in each particle size range could be calculated according to Equations (2) and (3).

(2) $$V_{p}=W_{p}/(\rho_{p}V)$$

(3) $$V_{p}[d_{s},d_{s+1}]=\frac{P(d_{s})-P(d_{s+1})}{P(d)-P(d_{\min})}\times V_{p}\times V$$

where *V_p_* is the volume proportion of the fine aggregate in mortar; *W_p_* is the total mass of the fine aggregate; *ρ_p_* is the apparent density of the fine aggregate; *V_p_*[*d_s_*, *d_s_*_+1_] is the volume of the fine aggregates with a particle size ranging from *d_s_* to *d_s_*_+1_; and *d*_min_ is the minimum particle size of the fine aggregates.

The Fuller grading curve is suitable for aggregate grading in three-dimensional (3D) space. Due to the huge calculation workload of the 3D mesoscale numerical simulation and the substantial calculation capacity requirements, the aggregate gradation conversion relationship between 3D space and a section of the specimen was established based on the Fuller grading curve and probability theory [41]. The aggregate grading curve was transformed from 3D space into a 2D plane, and the 2D aggregate gradation model was established. The probability of any point in the mortar being located inside the fine aggregate was regarded as its volume ratio *V_p_*, and then the probability function expression of a point located inside the fine aggregate with a particle size of d0 was obtained.

(4) $$P(d<d_{0})=V_{p}{\left(\frac{d_{0}}{d_{\max}}\right)}^{\frac{1}{2}}$$

By deriving and calculating Equation (4), the probability density function could be obtained, as shown in Equation (5).

(5) $$P^{\prime }(d_{0})=V_{p}\frac{\partial P({d<d}_{0})}{\partial d_{0}}=\frac{1}{2}V_{p}{\left(\frac{d_{0}}{d_{\max}}\right)}^{\frac{1}{2}}\times \frac{1}{d_{\max}}$$

According to Equations (4) and (5), the probability that a certain point in the 2D model was located in the fine aggregate with a diameter exceeding *d*_0_ could be obtained, as shown in Equation (6). Finally, the particle size distribution function of 2D fine aggregates could be obtained by Equation (7).

(6) $$P_{c}({d>d}_{0})=\int_{d_{0}}^{d_{\max}}\left[P^{\prime }(d_{0}\right)\times P_{d_{0}}({d>d}_{0})]dd_{0}$$

(7) $$\begin{array}{l} P_{c}({d>d}_{0})=V_{p}(1.065d_{0}^{0.5}d_{\max}^{-0.5}-0.053d_{0}^{4}d_{\max}^{-4}-0.012d_{0}^{6}d_{\max}^{-6}\\ -0.0045d_{0}^{8}d_{\max}^{-8}-0.0025d_{0}^{10}d_{\max}^{-10}) \end{array}$$

### 3.2. Random Generation of Fine Aggregates in Mortar

The random aggregate model of mortar could be generated by the Monte Carlo method [42]. Based on the theory of probability and statistics, the Monte Carlo method abstracts the problem to be solved as a random process model. The method determines the statistical characteristics of the parameter variables in the random process model by computer simulation and uses random numbers to solve the uncertainty problem. Under the boundary conditions and physical conditions, the Monte Carlo method was adopted to randomly position the fine aggregate, and then a random aggregate structure model of mortar was generated. In the process of random aggregate placement, the following conditions need to be met: (1) the fine aggregate should not exceed the boundary of the specimen; (2) there should be no overlap between fine aggregates; (3) the fine aggregates should meet the conditions of aggregate gradation and volume fractions; and (4) the input process of large-particle-size aggregates should occur prior to that of small-particle-size aggregates, so as to avoid the dispersion of small-particle-size aggregates occupying the overall space and affecting the feeding process of the large aggregates.

In the random aggregate model of mortar, the control parameters were only the circular coordinates and the radius when the aggregate was set as circular. The position function of the circular aggregate was the random number *P_i_*(*x_i_*, *y_i_*), and the particle size of the aggregate was *P_c_*[*d_s_*, *d_s_*_+1_]. If the chance of the fine aggregate appearing in the range of the mortar specimens was equal, then the probability density functions *f*(*x_i_*) and *f*(*y_i_*) of uniformly distributed random number *P_i_*(*x_i_*, *y_i_*) could be expressed by Equations (8) and (9), respectively.

(8) $$f(x_{i})=\left\{ \begin{array}{l} \frac{1}{x_{r}-x_{l}}\begin{array}{cc} & x\in [x_{r},x_{l}] \end{array}\\ \begin{array}{cc} & \begin{array}{ccc} 0 & & x\notin [x_{r},x_{l}] \end{array} \end{array} \end{array}\right.$$

(9) $$f(y_{i})=\left\{ \begin{array}{l} \frac{1}{y_{r}-y_{l}}\begin{array}{cc} & y\in [y_{r},y_{l}] \end{array}\\ \begin{array}{cc} & \begin{array}{ccc} 0 & & y\in [y_{r},y_{l}] \end{array} \end{array} \end{array}\right.$$

According to Equation (7), the probability density function *P_c_*[*d_s_*, *d_s_*_+1_] of aggregate particle size distribution with aggregate particle sizes between *d_s_* and *d_s_*_+1_ could be obtained as follows:

(10) $$P_{c}[d_{s},d_{s+1}]=P_{c}({d>d}_{s})-P_{c}({d>d}_{s+1})$$

**Figure 2:**
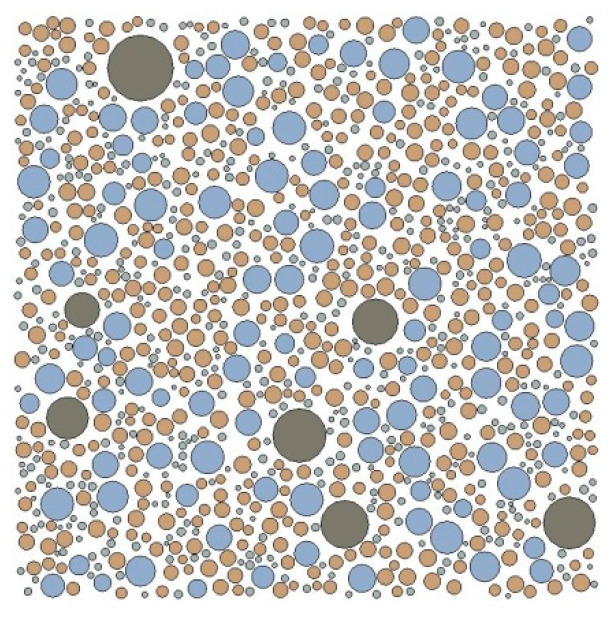
2D RCA structure of mortar.

Figure 2 shows a 2D random circular aggregate (RCA) structure of mortar generated by the Monte Carlo method, with a sample size of 20 mm × 20 mm.

The generation process of circular aggregates is simple, efficient, and easy to realize, but circular aggregates are obviously different to actual aggregates. Most actual aggregates have a long, flat shuttle or polygonal shape, and there are few circular aggregates. Therefore, it was necessary to improve the shape of the fine aggregates to bring it closer to the shape of actual aggregates. The real shape of fine aggregates in mortar is most commonly a random polygon. The randomly generated circular aggregates waere used as the base aggregates for convex polygon aggregates, and the centroid of the base aggregates was used as the location parameter of the convex polygonal aggregates to generate random polygonal aggregates. The centroid of the polygonal aggregates was consistent with that of the base aggregates, and the position parameters of the base aggregates were randomly generated in the mortar specimen, so it can be considered that the polygonal aggregates were randomly distributed in the mortar specimen. Subsequently, the polygon was regarded as the shape formed by connecting each vertex in turn, and the shape and area of the aggregates were controlled by the polygon vertex parameters. In this section, the algorithm for generating convex polygon aggregates was based on the random generation and packing algorithm for circular aggregates. The specific steps were as follows:

(1) Based on the random generation and packing algorithm for circular aggregates, the circular geometric characteristic parameters (*x*, *y*, *d*) were generated, and the center coordinates (*x*, *y*) were used as the position parameters of the polygonal aggregates.
(2) We used the random calculation module of the Python language in Abaqus software to randomly generate the number of vertices, *n*, of the polygon and solved Equation (11) to generate the polar coordinates of the first point (*r*, *θ*) on the circumference as the initial vertex of the polygon.
  (11) $$\left\{ \begin{array}{l} r=r_{0}\\ \theta =\frac{2\pi }{n}+(\lambda -1)\theta_{b} \end{array}\right.$$
  where *λ* is a random number within the range (0, 1) and *θ_b_* is the polar angle.
(3) The second to *n*th vertex coordinates (*r_i_*, *θ_i_*) were obtained by Equation (12).
  (12) $$\left\{ \begin{array}{l} r_{i}=r_{0}+(\lambda -1)r_{b}\\ \theta_{i}=\theta_{i+1}+\frac{2\pi }{n}+(\lambda -1)\theta_{b} \end{array}\right.$$
  where *r_b_* is the polar radius. If the value of the polar angle and polar radius is too large, it will lead to a fine aggregate with a needle shape. To avoid the generation of deformed fine aggregates, the value ranges of the polar angle and polar radius were set as follows: *θ_b_* was a random number within the range (0, 2π/*n*^2^), and *r_b_* was a random number within the range (0, 0.5*r*_0_).
(4) We connected the generated vertices in turn to form a polygon aggregate.

**Figure 3:**
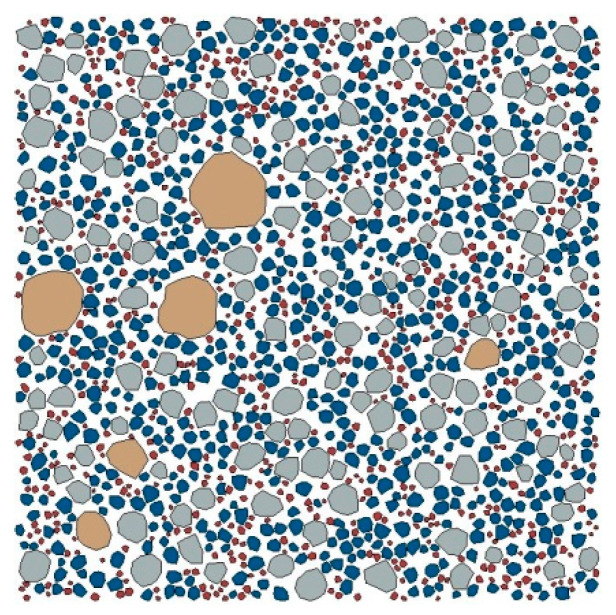
2D RPA structure of mortar.

Figure 3 shows a 2D random polygonal aggregate (RPA) structure of mortar generated by the Monte Carlo method, with a sample size of 20 mm × 20 mm.

## 4. Mesoscale FE Simulation of Corroded Mortar under Uniaxial Compression

In this section, the mortar specimen corroded in sodium sulfate solution was regarded as a three-phase composite of sand, cement paste, and ITZ. A 2D mesoscale FE model was established by Abaqus 6.14-5 software to simulate the uniaxial compression failure process of corroded mortar, including the establishment of a random aggregate model, the setting of boundary conditions, the selection of damage models, the acquisition of mechanical parameters, and the establishment of a constitutive model of the mesoscale phases.

### 4.1. Establishment and Grid Division of the Random Aggregate Model

On the mesoscale, the corroded mortar was regarded as a three-phase composite of sand, cement paste, and ITZ. The ITZ thickness of the mortar at 88 d was 20 μm [40]. In the simulation, if the ITZ thickness was taken as the actual thickness of 20 μm, the area around the ITZ needed to be arranged with a fine grid, which produced problems related to grid division and calculation efficiency. Considering the efficiency of computer calculation, it was difficult to take the micron scale as the thickness of the mortar ITZ. In previous mesoscale simulations of concrete, the area within the thickness range of 0.5 mm to 2 mm around the aggregate particles has often been set as the thickness of the ITZ [43,44]. In the mesoscale FE simulation of mortar, if the thickness of the ITZ was set to the millimeter order, the thickness of the ITZ and the particle size of the fine aggregates were in the same order of magnitude. This greatly increased the volume fraction of the ITZ, which was obviously inconsistent with the reality. To better consider the influence of the ITZ on the mechanical properties of the mortar, the ITZ and cement paste were combined to form an equivalent matrix, and the thickness of the ITZ was set to 20 μm according to [40]. On the mesoscale, the corroded mortar was simplified as a composite material of sand and the equivalent matrix, and a mesoscale FE model of corroded mortar, considering the influence of the ITZ, was established.

**Figure 4:**
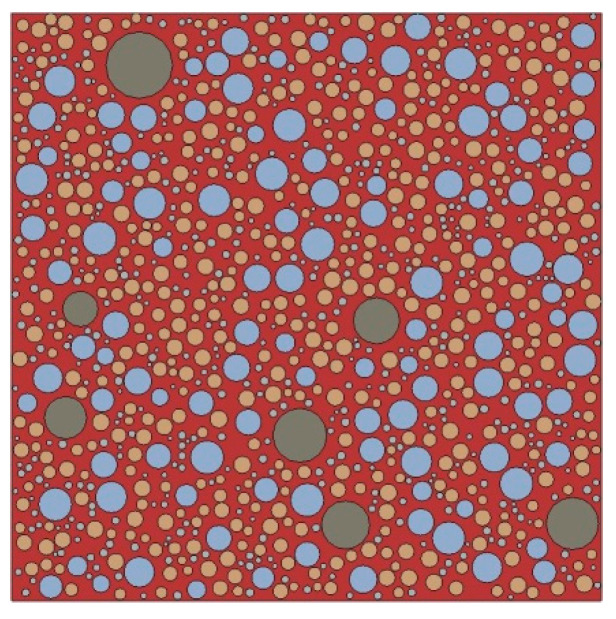
2D RCA model.

**Figure 5:**
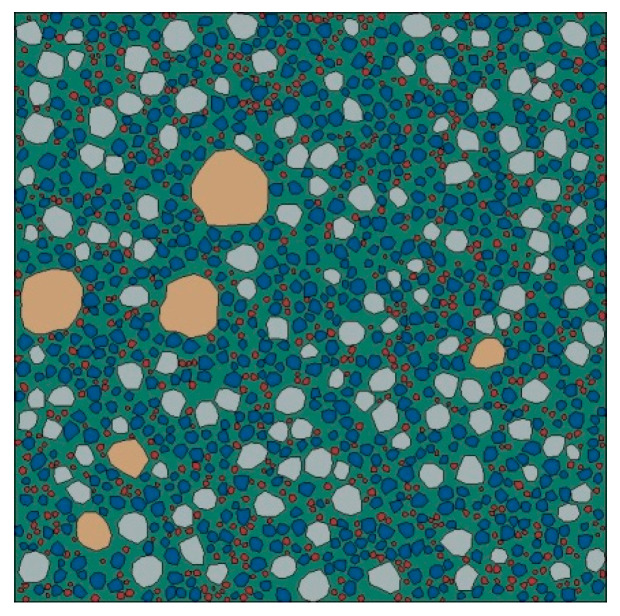
2D RPA model.

Based on the structural parameters of the fine aggregates obtained in Section 3, a 2D RCA model and a 2D RPA model of corroded mortar were established using Abaqus software, as shown in Figure 4 and Figure 5, respectively.

**Figure 6:**
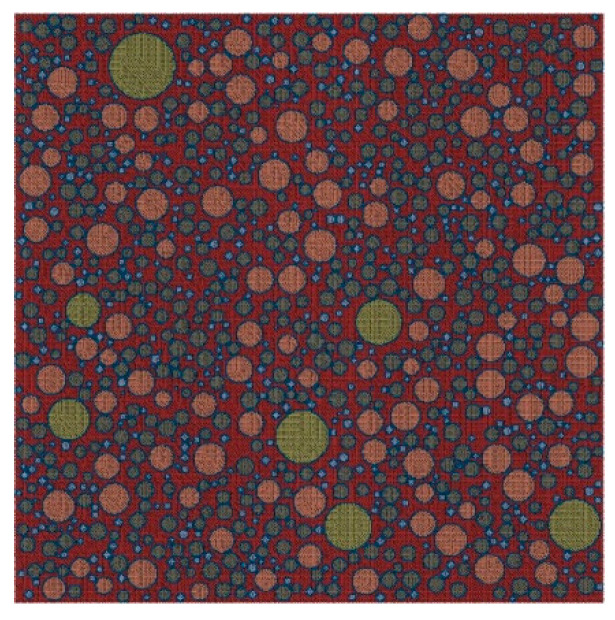
2D RCA model after meshing.

**Figure 7:**
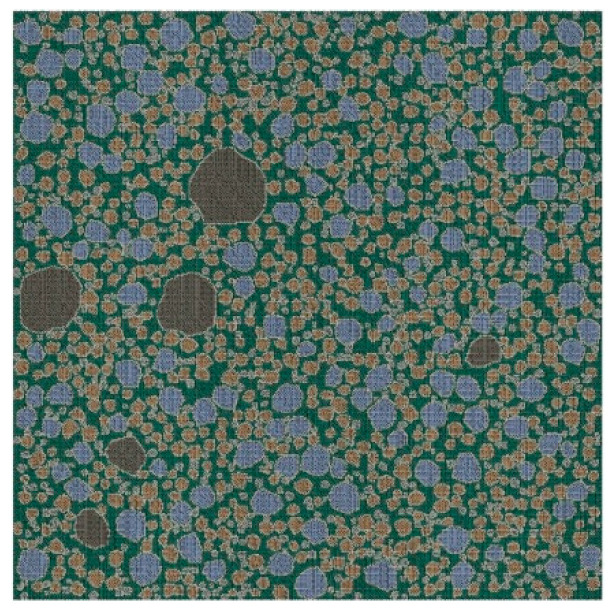
2D RPA model after meshing.

During the simulation process, if the parameters of the material properties, loads, and boundary conditions were directly defined on the elements and nodes, then the parameters had to be modified when the mesh of the FE model was modified. Therefore, the parameters were directly defined on the geometric model rather than on the elements and nodes. Sharp corners that were overly small appeared during the generation of the convex polygon aggregates, resulting in many refined meshes during the model meshing. Thus, the geometric model needed to be modified, leading to the failure of the defined loads and boundary conditions. Therefore, the loads and boundary conditions were set after the meshing of the element grid to avoid the influence of the geometric model modification. The free mesh of the random aggregate model was divided by a quad-dominated quadrilateral element. The mesh was based on a neutral axis algorithm with the element attribute of CPS4R and a mesh size of 0.05 mm. Figure 6 and Figure 7 show the 2D RCA model and 2D RPA model after meshing, respectively.

### 4.2. Selection of Damage Model and Determination of Boundary Conditions

The mortar material was a quasi-brittle material, so it was very important to describe the elastic and plastic properties of the material accurately. The concrete damaged plasticity model provides a general capacity for modeling concrete and other quasi-brittle materials using the concepts of isotropic damaged elasticity in combination with isotropic tensile and compressive plasticity, and it can be used to describe the elastic–plastic properties and the damage change law of the material. The model uses isotropic elastic damage combined with isotropic tensile and compressive plasticity to replace the inelastic behavior of a material, and it was suitable for the 2D mesoscale FE simulation of the mortar uniaxial compression process. Therefore, the concrete damaged plasticity model was selected to simulate the uniaxial compression behavior of the corroded mortar specimens in this paper.

**Figure 8:**
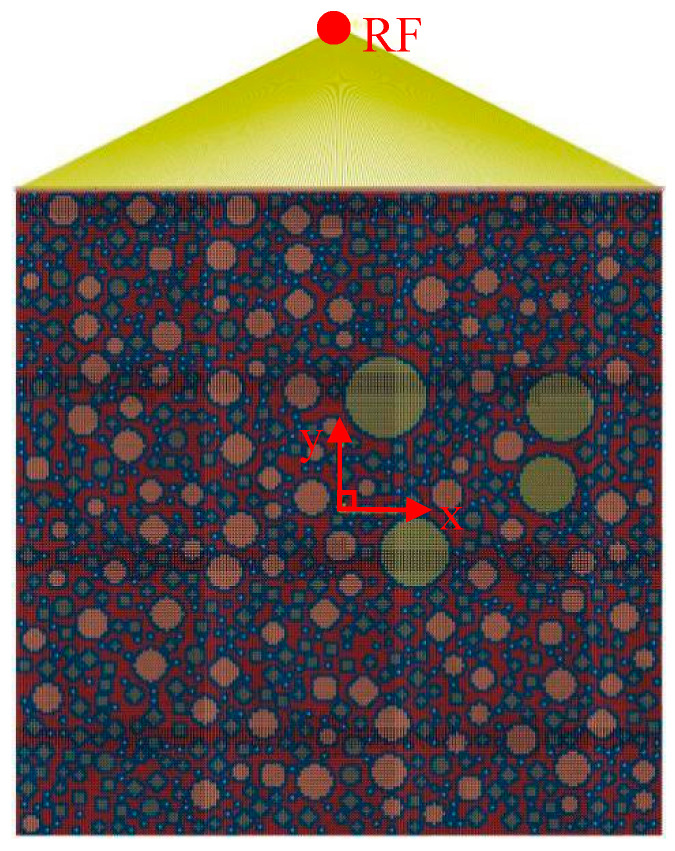
2D RCA model with coupling constraints.

**Figure 9:**
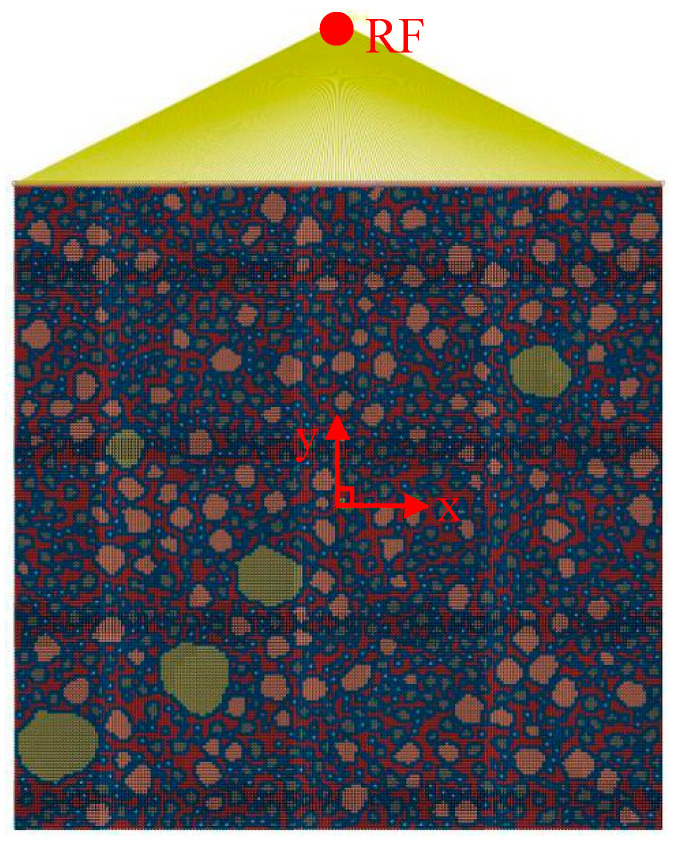
2D RPA model with coupling constraints.

To generate the 2D mesoscale FE simulation of the uniaxial compression process of the corroded mortar specimen, the 2D meshing model was imported into Abaqus software. The displacement of all nodes on the bottom surface of the corroded mortar specimen in the Y-axis direction was set as 0, and the rotation angles were set as free. The numerical simulation stress–strain curve of the 2D mesoscale model of the mortar uniaxial compression process was obtained by setting the reference point (RF). The displacement value of the RF in the Y direction was equal to the displacement value of the reference plane (the upper surface) in the Y direction. At the same time, the reaction force of the RF in the Y direction was the sum of the reaction forces of all nodes in the reference plane in the Y direction. By outputting the reaction force and displacement relation of the RF, it could be used to express the relation between the displacement and reaction force of the upper surface in the Y-axis direction. Then, the stress–strain curve of the 2D mesoscale model numerical simulation of the mortar uniaxial compression process could be obtained. The reference point (RF) was set at a certain distance from the center point of the reference surface (upper surface) in the positive direction of the Y-axis. The RF and all the nodes of the upper surface were coupled by Tie constraint binding. The displacements of the RF along the X-axis and the Z-axis were set as 0. In order to avoid the inclination of the upper surface of the mortar specimen, the rotation angles of the RF were set as 0. The distance between the RF and the upper surface had no effect on the output results. Hence, in this paper, the RF was set as 5 mm from the center point of the upper surface in the Y direction. The 2D RCA model and 2D RPA model of the mortar (M2) after the application of coupling constraints between the RF and the upper surface are shown in Figure 8 and Figure 9, respectively. According to the uniaxial compression test results, the mortar sample was obviously damaged when the strain was 1%. The length of the mortar sample was 20 mm; the strain value was 1% in the Y direction, and the loading displacement of the RF was calculated as 0.2 mm (20 × 1% = 0.2). In this paper, Abaqus software was used to numerically simulate the uniaxial compression process of the mortar specimens under sulfate corrosion. The RF was set as 5 mm from the center point of the upper surface in the Y direction, and the loading displacement was set as −0.2 mm.

### 4.3. Determination of Mechanical Parameters and Calibrated Constitutive Relations of Mesoscopic Phases

During the simulation, the constitutive relations and mechanical parameters (elastic modulus, Poisson’s ratio, and strength value) of the mesoscale phases in the corroded mortar had a great influence on the simulation results. The corroded mortar was regarded as a composite material of the equivalent matrix (cement paste and ITZ) and sand. The determination of the mechanical parameters and calibrated constitutive relations of the mesoscopic phases is described in this section.

#### 4.3.1. Acquisition of Elastic Modulus and Poisson’s Ratio

Under sulfate attack, the sulfate ions did not react with the sand, and the mechanical properties of the sand were assumed to be unchanged. During the uniaxial compression of corroded mortar, the sand was not damaged. Therefore, it was assumed that the sand was a linear elastomer. Guan [40] studied the elastic modulus of sand in corroded mortar (M1 and M2) at 88 d (with a corrosion time of 60 d using 5 wt% Na_2_SO_4_) by a nanoindentation test. The fluctuation range of the elastic modulus of the sand was relatively small, and the average elastic modulus of the sand in the corroded mortar was 63.25 GPa [40]. The Poisson’s ratio of the sand was 0.14.

The elastic parameters of the equivalent matrix in the corroded mortar were deduced by two-step homogenization methods [40]. The solution process of the elastic parameters was as follows: On the microscale, the corroded cement paste was regarded as a composite of 10 microphases [13]. Considering the influence of the erosion time and the spatial distribution of sulfate ions, the mechanical parameters and volume fractions of the microphases of the corroded cement paste were determined from the results of nanoindentation tests and the X-CT–hydration–deterioration model [40]. Then, the elasticity parameters of the cement paste at 88 d were calculated by the SC method. The shear modulus and bulk modulus of the cement paste (w/c = 0.53) were 14.03 GPa and 8.63 GPa, respectively, and the values of the cement paste (w/c = 0.35) were 15.13 GPa and 9.33 GPa, respectively. Subsequently, the volume fractions of the phases in the corroded mortar were calculated by the mortar mix proportion and Lu and Torquato’s model [45,46], and the results are shown in Table 4. According to the nanoindentation experiment, the elastic modulus ratio of the ITZ and cement at the same corrosion layer depth in the corroded mortar was 0.7 (M1) and 0.8 (M2). The elastic modulus ratio of the ITZ and matrix in the mortar remained constant. Combined with the elastic modulus of the corroded cement, the average elastic modulus of the ITZ in the corroded mortar (M1 and M2) was 15.2 GPa and 18.9 GPa, respectively. Based on the elastic parameters and volume fractions of the cement and ITZ, the elastic parameters of the equivalent matrix in the corroded mortar were calculated. The elastic modulus of the equivalent matrix in the corroded mortar (M1 and M2) was 19.99 GPa and 22.25 GPa, respectively. The Poisson’ ratio of the equivalent matrix in the corroded mortar was 0.24.

#### 4.3.2. Calibration of Strength

**Figure 10:**
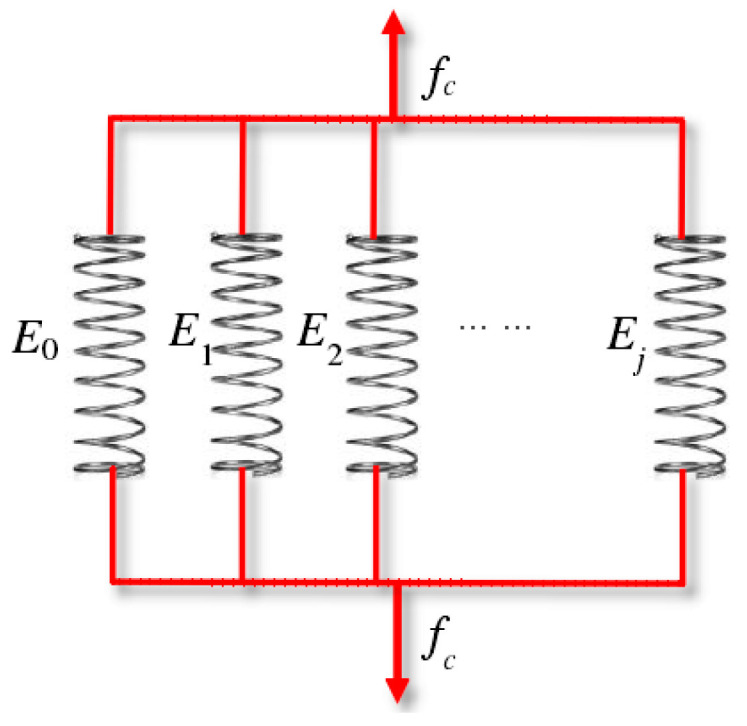
Schematic diagram of Voigt’s parallel model.

The compressive strength of the mesophases in the corroded mortar was calculated using Voigt’s parallel model. Voigt [47] first used the parallel model to study the effective elastic properties of heterogeneous composites. Then, the parallel model was used to study the mechanical behavior of cement-based materials on the micro/meso/macroscale. Li [48], Kandarpa [49], and Du [50,51] studied the mechanical behavior of concrete materials between the macroscale and microscale, including the elastic modulus, strength, and stress–strain curve. Subsequently, Li [30] studied the relationship between the strength values of micro-compositions and the strength values of the mesophases in cement paste under sulfate attack. In this study, the elements of the composite material were ideally simplified as a series of parallel micro/meso-elements under uniaxial loading. Both ends of the element were fixed on a rigid plate, and the deformations of the elements were kept the same, so that it satisfied the plane section assumption. Each element represented a micro-characteristic of the composite material, and the whole parallel system reflected the macro-mechanical properties of the composite material. Voigt’s parallel model, as shown in Figure 10, assumes that all of the micro/meso-components are parallel to the loading direction, meaning that the strain of all the components is in the loading direction and has a constant value, ensuring the deformation coordination of the material elements. The research results proved that the parallel model was feasible and appropriate for studying the macro-mechanical behavior of the composite material from the micro/mesoscale. Therefore, Voigt’s parallel model was an excellent tool for predicting the compressive strength of the composites.

According to Voigt’ parallel model [47], the strain of each component is the same as the average strain of the composite material. Thus, the average stress can be expressed as:

(13) $$f_{c}=\sum_{j=1}^{n}C_{j}f_{cj}$$

where

(14) $$f_{cj}=E_{j}\varepsilon_{j}=E_{c}\varepsilon_{j}$$

In the above equations, *f_c_* is the compressive strength of composites, *C_j_* is the volume fraction of component *j*, *f_cj_* is the compressive strength of component *j*, *E_j_* is the elastic modulus of component *j*, *ε_j_* is the strain of component *j*, and *E_c_* is the elastic modulus of the composites.

The compressive strength of the cement paste at 88d was 54.992 MPa (M1) and 60.54 MPa (M2) [13]. According to the plane section assumption and the minimum compressive strain criterion, the compressive strength values of the equivalent matrix in the M1 and M2 mortar could be obtained using the Voigt parallel model with the volume fractions, elastic modulus, and compressive strength of the ITZ and cement. The values were 48.28 MPa and 52.33 MPa, respectively. The tensile strength of the concrete material was 1/10 of the compressive strength [52]. Therefore, the tensile strength values of the equivalent matrix in the M1 and M2 mortar were 4.828 MPa and 5.233 MPa, respectively.

#### 4.3.3. Calibration of Constitutive Relations

The mechanical constitutive relations of the phases were an extremely important foundation for the mesoscale FE simulation. Up to now, there have been few studies on the application of cement paste constitutive relations. Mortar and concrete are heterogeneous and nonlinear quasi-brittle materials with similar physical and mechanical properties. According to the classic compressive and tensile constitutive relations of concrete in the specification (GB 50010-2010) [52], Guan [30] established the constitutive relations of corroded cement by the calibration of parameters such as the elastic stage and peak strain. Then, the calibrated constitutive relations were adopted to describe the mechanical behavior of the equivalent matrix.

According to the classic compressive constitutive relation of concrete in the specification (GB 50010-2010) [52], the calibrated compressive constitutive relation of the equivalent matrix under sulfate attack was established [30], and the expressions were as follows:

(15) $$y=\frac{2x}{1+x^{2}}\begin{array}{cc} , & 0.6\leq x<1 \end{array}$$

(16) $$y=\frac{x}{2.38{\left(x-1\right)}^{2}+x}\begin{array}{cc} , & x \end{array}\geq 1$$

(17) $$y=1.818x\begin{array}{cc} , & 0\leq x<0.6 \end{array}$$

**Figure 11:**
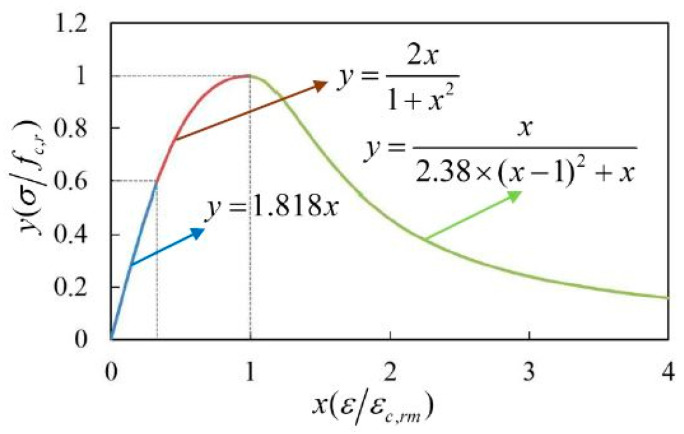
The calibrated compressive constitutive relation curve [30].

The calibrated compressive constitutive curve of the equivalent matrix in corroded mortar was obtained by Equations (15)–(17), as shown in Figure 11.

According to the classic tensile constitutive relation of concrete in the specification (GB 50010-2010) [52], the calibrated tensile constitutive relation of the equivalent matrix under sulfate attack was established [30], and the expressions were as follows:

(18) $$y=1.2x-0.2x^{6}\begin{array}{cc} , & 0.6\leq x<1 \end{array}$$

(19) $$y=\frac{x}{\alpha_{t}{\left(x-1\right)}^{1.7}+x}\begin{array}{cc} , & x\geq 1 \end{array}$$

(20) $$y=1.193x\begin{array}{cc} , & 0\leq x<0.6 \end{array}$$

(21) $$\alpha_{t}=0.312f_{t,r}^{2}$$

**Figure 12:**
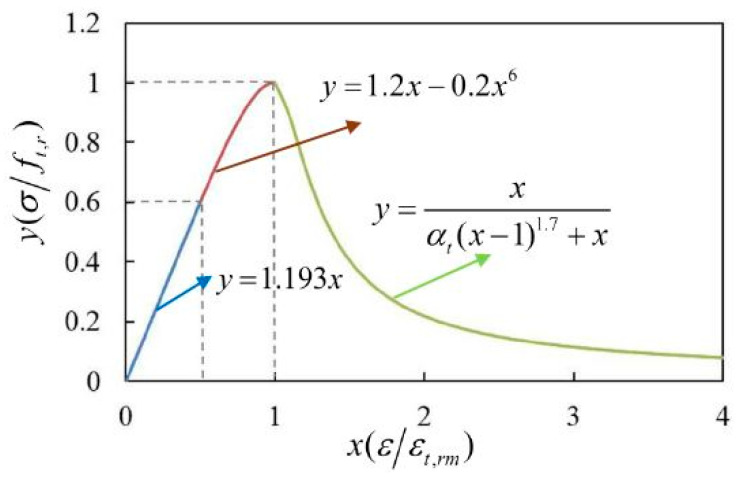
The calibrated tensile constitutive relation curve [30].

The calibrated tensile constitutive curve of the equivalent matrix in the corroded mortar was obtained by Equations (18)–(20), as shown in Figure 12.

## 5. Simulation Results and Analysis

### 5.1. Analysis of Uniaxial Compression Failure Process of High-Water/Cement-Ratio Mortar

**Figure 13:**
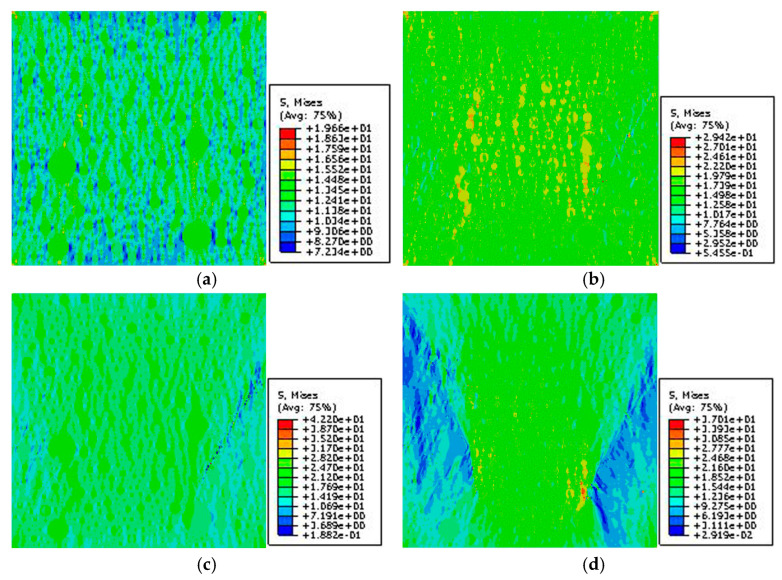
Stress nephogram of 2D RCA model for mortar (M1) with different strains: (**a**) ε = 0.03%; (**b**) ε = 0.1%; (**c**) ε = 0.3%; (**d**) ε = 0.45%.

**Figure 14:**
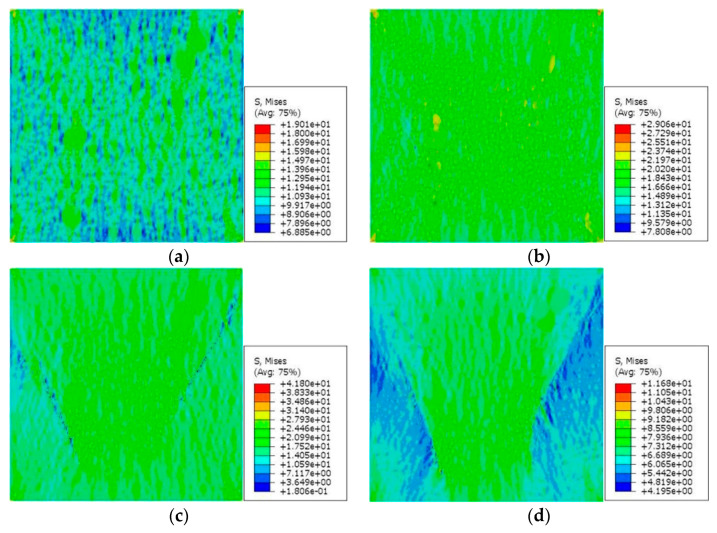
Stress nephogram of 2D RPA model for mortar (M1) with different strains: (**a**) ε = 0.03%; (**b**) ε = 0.1%; (**c**) ε = 0.3%; (**d**) ε = 0.45%.

Figure 13 and Figure 14 show the stress nephograms with different macrostrains during the uniaxial compression test simulation of the 2D RCA and 2D RPA models of corroded mortar (M1), respectively. On the mesoscale, the mortar was a composite material of sand and the equivalent matrix. Different constituent phases (sand and equivalent matrix) had different micromechanical properties, leading to the uneven stress distribution in the mortar specimen under uniaxial compression and resulting in the nonlinearity of the mortar.

As shown in Figure 13 and Figure 14, the 2D mesoscale model of corroded mortar was in a linear elastic stage at first. As regards the nonuniformity of the distribution and the difference in the mechanical properties of the mesoscale phases in the corroded mortar, the stress concentration and accumulated deformation appeared in the mortar with the increase in the compressive strain, resulting in the equivalent matrix units reaching the ultimate tensile stress of the material. Then, the 2D mesoscale model of corroded mortar entered the strain-softening stage, and the stiffness value decreased rapidly. The failure of the mortar sample was caused by the nonuniformity of the mesoscale distribution of the internal materials. Microcracks formed, propagated, and penetrated the material to create macrocracks. Meanwhile, the development of microcracks occurred in the equivalent matrix through the bypassing of the sand.

**Figure 15:**
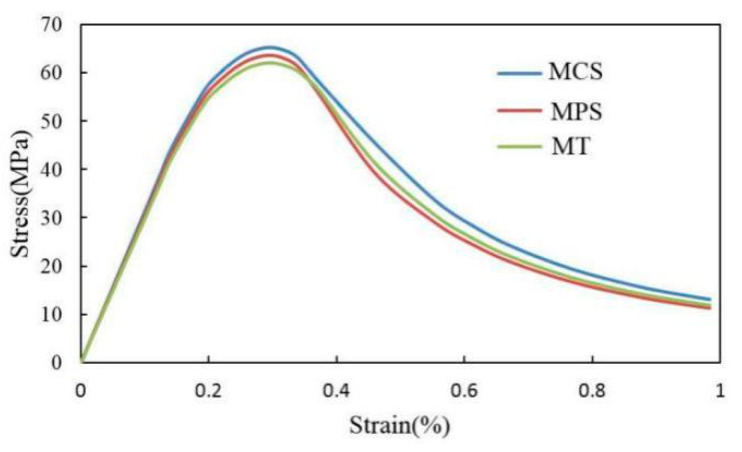
Uniaxial compression stress–strain curve of mortar (M1).

Figure 15 shows the stress–strain relation curves obtained from the mesoscale FE simulation of the uniaxial compression test with the 2D RCA and 2D RPA models of the corroded mortar (M1). The MCS (mortar circular simulation) represents the stress–strain relation curve simulated based on the 2D RCA model of mortar. The MPS (mortar polygon simulation) represents the stress–strain relation curve simulated based on the 2D RPA model of mortar. The MT (mortar test) is the stress–strain relation curve obtained from the uniaxial compression test. In Figure 15, the stress–strain curves obtained by numerical simulations and experiments include a rising section, peak section, and falling section.

**Figure 16:**
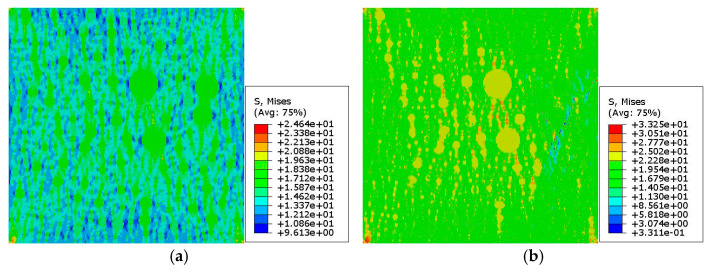

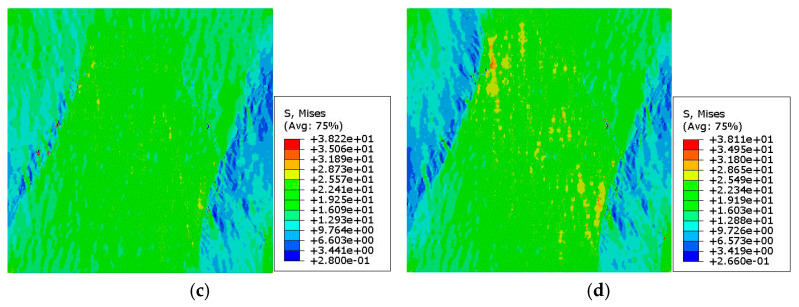
Stress nephogram of 2D RCA model for mortar (M2) with different strains: (**a**) ε = 0.03%; (**b**) ε = 0.1%; (**c**) ε = 0.3%; (**d**) ε = 0.45%.

By comparing the three types of stress–strain curve in Figure 16, it can be seen that the MPS curve is closer to the MT curve than the MCS curve, which shows that the 2D RPA model was closer to the real aggregate model than the 2D RCA model. In the mesoscale simulation, compared with the circular aggregate, the polygonal aggregate more easily replicated the phenomenon of stress concentration because the edge of the aggregate was more irregular, so the peak strength of the 2D RPA model was lower than that of the 2D RCA model. In the mesoscale FE simulation, the mortar was regarded as a two-phase composite of sand and the equivalent matrix, and the generation and development of cracks occurred in the equivalent matrix. However, in the real uniaxial compression test, the mortar was a multiphase composite of sand, cement paste, and ITZ, and the generation and development of cracks occurred in the ITZ. Compared with the equivalent matrix, the mechanical properties of the ITZ were obviously weaker. It can be observed that the peak stress of the MCS curve (65.25 MPa) and MPS curve (63.68 MPa) was higher than that of the MT curve (62.13 MPa), and the error values between these figures and the experimental peak stress were 5.0% and 2.5%, respectively.

### 5.2. Analysis of Uniaxial Compression Failure Process of Low-Water/Cement-Ratio Mortar

**Figure 17:**
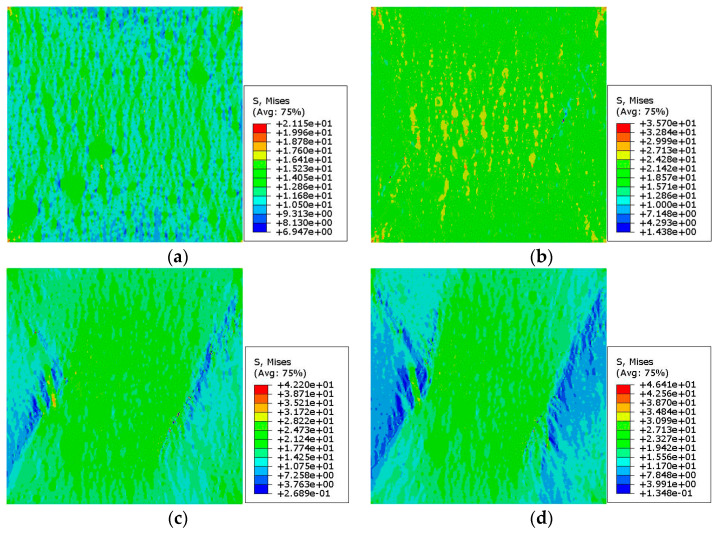
Stress nephogram of 2D RPA model for mortar (M2) with different strains: (**a**) ε = 0.03%; (**b**) ε = 0.1%; (**c**) ε = 0.3%; (**d**) ε = 0.45%.

Figure 16 and Figure 17 show the stress nephograms under different macrostrains during the uniaxial compression test simulation of the 2D RCA and 2D RPA models of the corroded mortar (M2), respectively. It can be seen that the mortar model was in the linear elastic stage when the compressive strain was small (ε = 0.03%). With the increase in the compressive strain, the accumulated deformation and stress in the mortar model increased gradually. As to the ultimate tensile strength of the equivalent matrix is far lower than that of the fine aggregates, the mesounits of the equivalent matrix are destroyed and cease functioning when the stress exceeds the ultimate tensile stress of the equivalent matrix. Some mesounits were damaged and microcracks were formed with the compressive strain of ε = 0.3%, as shown in the blue parts of Figure 16c and Figure 17c. With the continuous increase in the compressive strain (ε = 0.45%), a large number of mesounits were destroyed, and cracks continued to expand, penetrate, and produce macrocracks, as shown in the blue parts of Figure 16d and Figure 17d. The failure of the corroded mortar was a process of microcrack initiation, propagation, penetration, and progression into macrocracks in the equivalent matrix, which eventually led to the instability failure of the whole mortar specimen.

**Figure 18:**
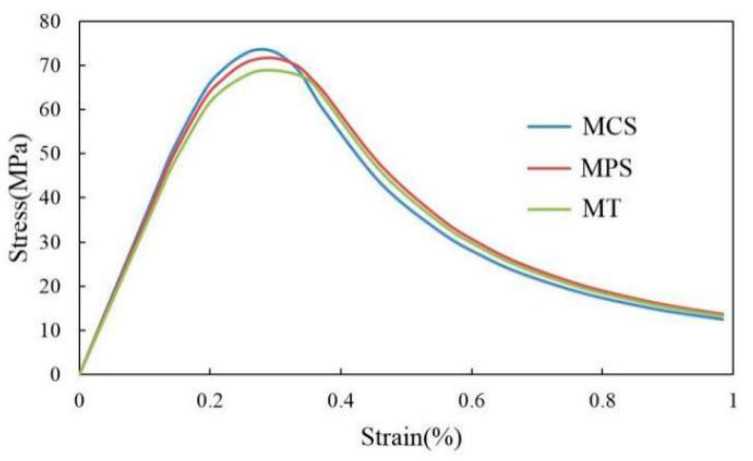
Uniaxial compression stress–strain curve of mortar (M2).

Figure 18 shows the stress–strain relation curves obtained from the mesoscale FE simulation of the uniaxial compression test with the 2D RCA and 2D RPA models of the corroded mortar (M2). The three types of stress–strain curve in Figure 18 were relatively similar, and the peak stress error values between the MCS curve (73.5 MPa) and MT curve (68.9 MPa) and between the MPS curve (71.6 MPa) and MT curve (68.9 MPa) were 6.7% and 3.9%, respectively. The error values were within 7%, which indicates that the mesoscale FE model of corroded mortar, considering the influence of the ITZ and based on the random aggregate model, could accurately simulate the uniaxial compression behavior of mortar under sulfate attack. Meanwhile, the MPS curve was closer to the MT curve than the MCS curve, which shows that the 2D RPA model was closer to the real aggregate model than the 2D RCA model.

## 6. Conclusions

A 2D mesoscale FE numerical model of mortar, considering the influence of the ITZ, was established to evaluate the uniaxial compression process of mortar corroded in 5 wt% sodium sulfate. Homogenization methods and calibrated relations were used to calibrate the input parameters of the model. Lastly, the simulation results were compared with the measured results of the uniaxial compression test, and the following conclusions were drawn:

(1) The failure of corroded mortar was caused by the nonuniformity of the mesoscale distribution of the internal materials. The formation and development of microcracks occurred in the equivalent matrix through the bypassing of the sand.
(2) The simulation results were in good agreement with the experimental results, which proves that the homogenization methods and the calibrated constitutive relations were effective.
(3) The MCS and MPS curves were close to the MT curve, and the error values of peak stress between the simulation results and the measured results were within 7%, indicating that the 2D mesoscale FE model could accurately simulate the uniaxial compression behavior of corroded mortar.
(4) The 2D RPA model of mortar was closer to the real aggregate model than the 2D RCA model.

## Figures and Tables

**Table 1:** Mineral composition of cement (%).

Composition	C_3_S	C_2_S	C_4_AF	C_3_A
Content	63.94	16.79	11.86	7.41

**Table 2:** Cumulative percentage retained of fine aggregates.

Particle size (mm)	4.75	2.36	1.18	0.6	0.3	0.15	0
Percentage (%)	1.6	4.0	8.81	49.29	91.57	96.78	100

**Table 3:** Mixture proportions of mortar.

Type	w/c	Water	Cement	Sand
M1	0.53	400	750	1500
M2	0.35	312.2	900	1350

**Table 4:** Volume fractions of phases in corroded mortar (%).

Type	Sand	Cement Paste	ITZ
M1	47.0	41.9	11.1
M2	46.0	43.1	10.9

## Data Availability

Not applicable.
